# Short-Chain Fatty Acids Regulate Poultry Feed Intake via the Hypothalamus: Receptor-Mediated and Epigenetic Mechanisms

**DOI:** 10.3390/ani16060954

**Published:** 2026-03-18

**Authors:** Yanli Wang, Xueqing Xiao, Bo Zheng, Dongying Bai, Yi Zhang, Wenrui Zhen, Bingkun Zhang, Yanbo Ma

**Affiliations:** 1 Department of Animal Physiology, College of Animal Science and Technology, Henan University of Science and Technology, Luoyang 471000, China; wangyanli@stu.haust.edu.cn (Y.W.); xueqingxiao@stu.haust.edu.cn (X.X.); zhengbo@stu.haust.edu.cn (B.Z.); 9900427@haust.edu.cn (D.B.); zhenwenr@126.com (W.Z.); 2 Henan International Joint Laboratory of Animal Welfare and Health Breeding, College of Animal Science and Technology, Henan University of Science and Technology, Luoyang 471000, China; 3 State Key Laboratory of Animal Nutrition, Department of Animal Nutrition and Feed Science, College of Animal Science and Technology, China Agricultural University, Beijing 100193, China; bingkunzhang@126.com; 4 Innovative Research Team of Livestock Intelligent Breeding and Equipment, Longmen Laboratory, Luoyang 471000, China

**Keywords:** short-chain fatty acids, microbiota–gut–brain axis, hypothalamus, feed intake regulation, neuroimmunity, poultry nutrition, precision feeding

## Abstract

The global poultry industry faces escalating challenges in animal welfare and production efficiency in the post-antibiotic era. Feed intake, a crucial determinant of growth, is frequently suppressed under stress, forming a key bottleneck for sustainable production. The microbiota–gut–brain (MGB) axis provides a novel framework to understand this complex regulation. Short-chain fatty acids (SCFAs), produced from dietary fiber by gut microbiota, serve as vital chemical messengers within this axis, modulating hypothalamic feeding centers and systemic metabolism. This review summarizes SCFA biosynthesis and kinetics in poultry and examines their neural, humoral, and immune pathways to the brain. Emphasis is placed on receptor-mediated signaling, epigenetic regulation, energy sensing, and neuroimmune modulation through which SCFAs regulate feeding and reduce inflammation. Practical strategies—such as substrate engineering, microbiota modulation, and exogenous regulators—are discussed to enhance SCFA levels and poultry health. Future directions include multi-omics integration, novel additives, and predictive models to advance antibiotic-free nutrition.

## Simple Summary

This review examines the central role of short-chain fatty acids (SCFAs) in regulating feed intake and health in poultry through the gut–brain axis SCFAs produced by gut microbes from dietary fiber, which act as chemical messengers that transmit signals from the intestine to the hypothalamic feeding center through neural circulatory and immune pathways. They help modulate appetite, reduce brain inflammation, and support metabolic balance. The article also discusses practical feeding strategies such as targeted fiber diets, probiotics, and plant-based compounds to enhance SCFA levels, providing science-based approaches to promote poultry health and productivity in antibiotic-free farming systems.

## 1. Introduction

### 1.1. Emerging Challenges in Poultry Production: Towards Comprehensive Lifetime Health Management Beyond Growth Performance

The global poultry industry is rapidly evolving towards intensification and large-scale production. Concurrently, the comprehensive implementation of antibiotic bans marks its official entry into the post-antibiotic era [1]. Within this context, traditional farming models focused solely on maximizing growth performance indicators (e.g., average daily gain, feed conversion ratio) are increasingly inadequate to meet the industry’s growing demands for sustainability, animal welfare, and food safety [2]. Consequently, establishing and implementing a holistic health management system that spans the entire lifespan of poultry, from hatch to market (or culling), has emerged as a major challenge [3].

Under practical farming conditions, poultry are highly susceptible to various stressors, including environmental challenges (e.g., temperature fluctuations, high stocking density), nutritional imbalances, and pathogen infections [4]. These challenges often lead to a range of health issues, including impaired gut barrier function, compromised immune competence, and metabolic disorders, which substantially impair growth performance, product quality, and ultimately, economic returns [5]. Notably, the significant reduction in feed intake observed in stressed poultry is of particular concern. Feed intake suppression not only directly reduces nutrient intake and utilization efficiency but also further weakens immune function, creating a vicious cycle of “reduced intake–immune suppression–disease susceptibility” that significantly increases farming risks [6].

Therefore, the modern poultry industry urgently needs to transcend traditional health management concepts and develop integrated strategies that address multiple dimensions, such as growth performance, immune function, gut health, metabolic balance, and animal welfare. This holistic approach is essential to address the myriad challenges of the post-antibiotic era and achieve a green, efficient, and sustainable poultry industry [7].

### 1.2. The Rise of the Microbiota–Gut–Brain Axis: Definition, Components, and Its Central Role in Stress Response and Behavioral Regulation

The “microbiota–gut–brain axis” (MGB axis) has become a research hotspot in life sciences and medicine in recent years. Its conceptualization and development have provided a novel, systemic perspective for understanding integrated physiological functions and behavioral regulation [8]. By definition, the MGB axis is a complex, bidirectional communication network between the gut microbiota and the central nervous system (CNS), mediated through neural, humoral, immune, and other pathways [9]. Core components of this system include the gut microbiota; the gut barrier system (encompassing physical, chemical, biological, and immune barriers); the autonomic nervous system (particularly the vagus nerve); the central nervous system (including key brain regions such as the hypothalamus and brainstem); and the endocrine system (e.g., enteroendocrine cells and their secreted hormones) [10].

In stress response regulation, the MGB axis acts as a “first responder.” When poultry encounter external stressors (e.g., heat, crowding), the composition and structure of their gut microbiota undergo rapid changes, leading to dysbiosis [11]. This dysbiosis can further compromise intestinal barrier integrity, increase gut permeability, and allow gut-derived endotoxins (e.g., lipopolysaccharide, LPS) and other harmful metabolites to enter the systemic circulation, thereby triggering a systemic low-grade inflammatory response [12].

Simultaneously, the altered gut microbiota can transmit stress signals directly to the CNS via vagal afferent nerve endings in the gut, or indirectly modulate the activity of the hypothalamic–pituitary–adrenal (HPA) axis, the core of the central stress response, by regulating the secretion of neuroactive substances such as serotonin (5-HT) and cholecystokinin (CCK) from enteroendocrine cells, thereby finely tuning the intensity and duration of the stress response [13,14].

Regarding behavioral regulation, substantial evidence indicates that the MGB axis plays a crucial role in modulating various behavioral phenotypes in poultry, including feeding behavior, exploratory behavior, social behavior, and fear responses [15]. Among these, feeding behavior, as a fundamental activity for nutrient acquisition and survival, is closely linked to MGB axis function. Metabolites produced by the gut microbiota from dietary fiber, such as SCFAs, can act on central feeding regulatory centers in the CNS (e.g., the hypothalamus) via multiple pathways, influencing the expression and secretion of neuropeptides (e.g., neuropeptide Y, NPY; pro-opiomelanocortin, *POMC*), thereby precisely regulating feeding motivation, feed intake, and feeding rhythms in poultry [16].

Furthermore, the gut microbiota can influence emotional states and cognition-related behaviors in poultry by modulating the levels of key neurotransmitters such as dopamine (DA) and γ-aminobutyric acid (GABA) in the CNS, consequently affecting overall welfare [17,18]. Thus, the MGB axis holds a central position in stress response and behavioral regulation, and in-depth investigation of its mechanisms is of significant theoretical and practical value for elucidating the intrinsic principles governing poultry health and behavior.

### 1.3. Core Focus of This Review: SCFAs as Indispensable Chemical Messengers in the MGB Axis, Bridging Dietary Nutrition and Brain Function Output

Within the complex bidirectional communication network of the MGB axis, the transmission and decoding of signaling molecules are key to its function. Short-chain fatty acids, primarily acetate, propionate, and butyrate, are core end-products of dietary non-starch polysaccharide (dietary fiber) fermentation by the gut microbiota. Owing to their unique chemical properties and broad biological activities, SCFAs have emerged as indispensable “chemical messengers” or a form of “molecular language” within the MGB axis, effectively bridging front-end dietary nutrition with terminal brain function and behavioral output [19].

From the perspective of front-end dietary nutrition, diet composition and structure, particularly the physicochemical properties of dietary fiber such as type, content, solubility, and degree of polymerization, directly determine the fermentation substrate available for the gut microbiota. This, in turn, profoundly influences total SCFA production, the proportional representation of individual SCFAs (the SCFA profile), and their spatial distribution along the digestive tract [20,21].

For instance, soluble dietary fibers (e.g., pectin, β-glucans, inulin) are readily and rapidly fermented by gut microbes, efficiently generating SCFAs and often increasing the proportion of butyrate. In contrast, insoluble dietary fibers (e.g., cellulose, lignin) ferment more slowly, resulting in relatively lower total SCFA production, predominantly acetate; nevertheless, they play a vital role in maintaining normal gut physical structure and motility [22]. Therefore, dietary nutrition directly shapes the “vocabulary” (yield) and “syntax” (profile) of SCFAs as chemical messengers by precisely regulating the gut microbial fermentation process.

Regarding terminal brain function and behavioral output, SCFAs, as key chemical messengers, can efficiently convey information about the metabolic status of the gut microbiota to the central nervous system via multiple transmission pathways, neural (vagus nerve), humoral (blood circulation), and immune (cytokines), as will be detailed in this review [23]. In terms of brain function modulation, SCFAs can influence neurotransmitter synthesis and release, the initiation and progression of neuroinflammation, and the growth and differentiation of neural cells by acting on neurons and glial cells in the CNS, thereby maintaining normal physiological function and homeostasis of the CNS [24].

Concerning behavioral output, SCFAs play a particularly important role in regulating feeding behavior in poultry. They can modulate the expression and secretion of key neuropeptides (e.g., neuropeptide Y, *NPY*; pro-opiomelanocortin, *POMC*) in the hypothalamic feeding regulation center, enabling precise control over feeding motivation, intake volume, and feeding rhythm [25]. Additionally, SCFAs may indirectly influence exploratory and social behaviors in poultry by modulating the function of brain regions associated with emotion and cognition, thereby comprehensively affecting production performance and welfare [26].

In summary, SCFAs serve as key “chemical messengers” within the MGB axis, effectively bridging front-end dietary nutrition and terminal brain function and behavioral output. A comprehensive understanding of the mechanisms underlying SCFA action within the MGB axis is therefore of paramount importance for elucidating the intrinsic links between poultry health and nutritional regulation, which forms the central theme of this review.

It is important to acknowledge that, due to the limited availability of direct evidence in avian species—particularly regarding detailed molecular mechanisms within the hypothalamus—this review draws upon findings from mammalian studies (primarily rodents and humans) to construct a conceptual framework. Where such extrapolation occurs, it is explicitly noted throughout the text, and the need for direct validation in poultry is emphasized. This approach is necessary to advance the field while awaiting species-specific confirmation.

## 2. The Lifecycle of SCFAs: From Gut Fermentation to Systemic Messengers

### 2.1. Generation Map: Core Microbiota and Functional Genes Responsible for SCFA Production in the Poultry Gastrointestinal Tract (With Emphasis on the Cecum)

The poultry gastrointestinal tract is a complex micro-ecosystem. Its distinct segments (e.g., crop, proventriculus, gizzard, small intestine, cecum, colon) host unique microbial community structures due to differences in physiology, pH, oxygen concentration, and nutrient composition [27]. The cecum, as the most active site of microbial fermentation, possesses a large surface area and prolonged chyme retention time, providing an ideal environment for anaerobic microorganisms. Consequently, it serves as the primary site for SCFA production [28,29].

Within the cecum, the core microbiota involved in SCFA generation primarily belong to the phyla *Bacteroidetes* and *Firmicutes*, with minor contributions from Actinobacteria and Proteobacteria [30]. Key functional genera include Clostridium clusters XIVa and IV, Lactobacillus, and Eubacterium from *Firmicutes*, as well as Bacteroides from *Bacteroidetes* [31,32]. These microbial groups harbor specific functional genes encoding a suite of key enzymes involved in carbohydrate fermentation metabolism, enabling the breakdown and transformation of dietary carbohydrates indigestible by the host (primarily dietary fiber) into SCFAs—mainly acetate, propionate, and butyrate—which typically constitute approximately 90–95% of total SCFAs [33].

At the molecular level, SCFA synthesis involves several key metabolic pathways and functional genes [34,35]:

Acetate production: Pyruvate is converted to acetyl-CoA by the pyruvate dehydrogenase complex (encoded by the *pdhA*, *pdhB*, *pdhC*, and *pdhD* genes). Acetyl-CoA is then catalyzed by phosphotransacetylase (encoded by *pta*) and acetate kinase (encoded by *ackA*) to form acetate. The Wood–Ljungdahl pathway represents another important route for acetate generation in certain strict anaerobes.

Propionate production: Two main pathways exist. The first is the succinate pathway, involving phosphoenolpyruvate carboxylase (encoded by *ppc*), malate dehydrogenase (*mdh*), and a series of subsequent reactions, ultimately generating propionyl-CoA, which is converted to propionate. The second is the acrylate pathway, in which lactate is converted to lactyl-CoA by lactate dehydrogenase (*ldh*), followed by enzymatic reactions including lactyl-CoA dehydratase, leading to propionate production.

Butyrate production: Two acetyl-CoA molecules condense under the action of thiolase (encoded by *thl*) to form acetoacetyl-CoA. This is followed by a series of reactions involving β-hydroxybutyryl-CoA dehydrogenase and crotonase (encoded by *etfA* and *etfB*). Finally, butyryl-CoA:acetate CoA-transferase (encoded by *but*) transfers the CoA moiety to an acetate molecule to yield butyrate; alternatively, butyrate kinase (encoded by *buk*) can catalyze butyrate formation.

These core microbiota and their functional genes collectively form the molecular foundation for SCFA generation in the poultry cecum. Alterations in microbial community composition and gene expression levels directly influence the efficiency of SCFA production and the SCFA profile [36].

### 2.2. Fate and Distribution: SCFA Absorption, Portal Vein Transport, Tissue Metabolism, and Systemic Distribution

Following their generation in the gut lumen, the processes of absorption, transport, metabolism, and tissue distribution critically influence the scope and intensity of SCFA biological functions [37]. Because they are partially dissociated at physiological pH, SCFAs are absorbed through both passive diffusion and carrier-mediated transport. Non-dissociated SCFA molecules are relatively lipophilic and can cross enterocyte membranes via passive diffusion, a mechanism present throughout the intestine but more prominent in proximal segments [38].

In the distal gut (cecum and colon), the apical membrane of enterocytes highly expresses specific SCFA transporters, primarily monocarboxylate transporter 1 (MCT1, encoded by *SLC16A1*) and sodium-coupled monocarboxylate transporter 1 (SMCT1, encoded by *SLC5A8*). These transporters facilitate the efficient uptake of SCFAs from the gut lumen into enterocytes via active or secondary active transport [39,40]. Notably, the metabolic fate differs among SCFAs. Butyrate, owing to its higher lipophilicity and its role as the preferred energy source for colonocytes, is largely metabolized locally via β-oxidation within enterocytes at its site of production, supplying approximately 60–70% of their energy requirements [41]. In contrast, acetate and propionate are predominantly transported via the portal vein circulation.

SCFAs entering the portal blood, primarily acetate and propionate, are first transported to the liver. In the liver, propionate serves as a major substrate for gluconeogenesis. Through the action of key enzymes such as propionyl-CoA carboxylase and methylmalonyl-CoA mutase, it can be converted into glucose, thereby contributing to systemic energy homeostasis [42]. Acetate, by contrast, largely bypasses hepatic metabolism and enters the systemic circulation, where it is delivered to peripheral tissues including the heart, skeletal muscle, and adipose tissue. In these tissues, acetate can be converted to acetyl-CoA via acetyl-CoA synthetase, subsequently entering the tricarboxylic acid (TCA) cycle for oxidative energy production or serving as a substrate for de novo lipogenesis in the liver and adipose tissue [43].

A proportion of SCFAs, particularly acetate, can cross the blood–brain barrier (BBB) and enter the central nervous system. Due to its small molecular size and moderate lipophilicity, acetate traverses the BBB relatively efficiently via passive diffusion and MCT-mediated transport [44]. Upon entering the brain, acetate is primarily taken up by astrocytes and converted to acetyl-CoA by acetyl-CoA synthetase, serving both as an energy substrate for neural cells and as a precursor for the synthesis of the neurotransmitter acetylcholine [45].

The ability of propionate and butyrate to cross the BBB is comparatively limited; however, under specific conditions (e.g., increased BBB permeability), they may enter the CNS in small amounts and exert signaling functions, such as the histone deacetylase (HDAC) inhibitory activity of butyrate [46].

Regarding systemic distribution, SCFA concentrations exhibit pronounced gradients across tissue compartments. The highest concentrations are detected in cecal contents (approximately 50–150 mM). In portal vein blood, concentrations decline to several hundred micromolar to 1–2 mM, whereas in peripheral arterial blood, owing to hepatic first-pass metabolism and peripheral tissue utilization, SCFA levels are further reduced to approximately 50–200 μM [47]. These characteristics of absorption, transport, metabolism, and distribution provide the material basis for SCFAs to exert both local (intestinal) and systemic (hepatic, peripheral, and central) biological effects.

### 2.3. Concentration Kinetics: Dynamic SCFA Levels in Poultry and Influencing Factors (Age, Diet, and Environment)

**Figure 1:**
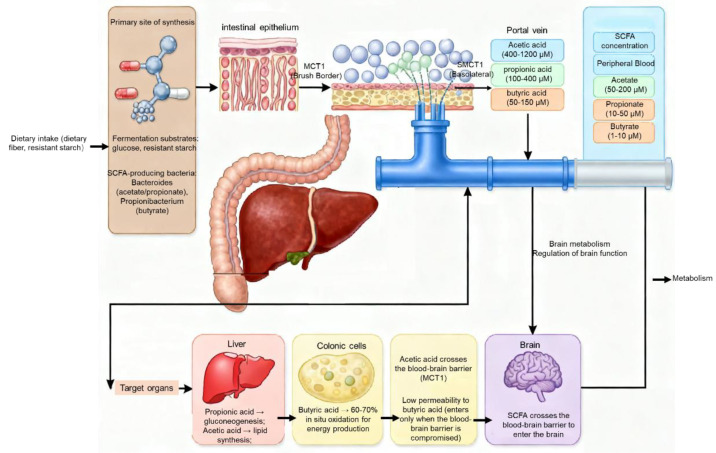
SCFA Life Cycle: Synthesis–Absorption–Systemic Distribution. The diagram illustrates the complete lifecycle of SCFAs in poultry: Synthesis in the cecum—dietary fiber is fermented by core microbiota (primarily *Bacteroidetes* and *Firmicutes*) via specific functional genes (e.g., pta, *ackA*, but) to produce acetate (50–70%), propionate (15–25%), and butyrate (10–20%). Absorption across the intestinal epithelium—SCFAs are absorbed via passive diffusion and carrier-mediated transport (MCT1, SMCT1). Butyrate is largely metabolized by colonocytes (∼60–70%) as their primary energy source, while acetate and propionate enter the portal vein. Systemic distribution—propionate undergoes extensive hepatic extraction (∼90%) for gluconeogenesis; acetate largely bypasses the liver and enters systemic circulation, reaching peripheral tissues (heart, muscle, adipose) and crossing the blood–brain barrier via MCT1-mediated transport and passive diffusion to access the hypothalamus. Concentration gradients (mM in cecum, μM in peripheral blood) are indicated.

SCFA levels in poultry are not static but exhibit complex dynamic fluctuations influenced by a combination of age, diet, and environmental factors [48] (Figure 1).

Age is a major intrinsic determinant of SCFA kinetics. At hatch, the chick intestine is nearly sterile, and SCFA production is minimal. With environmental exposure and the initiation of feeding, the gut microbiota rapidly colonize and undergo successional development. During the first 1–2 weeks post-hatch, facultative anaerobes (e.g., Lactobacillus) predominate, resulting in low total SCFA concentrations, primarily acetate [49]. As birds mature to 3–6 weeks of age, obligate anaerobes (e.g., Bacteroides and butyrate-producing Clostridia) gradually increase and become dominant. Concurrently, gut fermentation capacity matures, leading to a marked rise in total SCFA concentrations and an increased proportion of propionate and butyrate [50]. During later growth stages (e.g., the finishing phase) or the laying period, the gut microbiota structure stabilizes, and SCFA levels are maintained at relatively high and stable concentrations to support energy demands and gut health maintenance [51].

Dietary factors constitute key external regulators of SCFA kinetics. The content and source of dietary fiber directly determine the availability of fermentation substrates. Moderate increases in dietary fiber supply sufficient substrates for microbial fermentation, significantly enhancing total SCFA production [52]. However, excessively high fiber levels may promote over-fermentation and excessive gas production, potentially causing bloating and indigestion and thereby disrupting SCFA homeostasis [53]. Importantly, fiber type exerts a profound influence on the SCFA profile. Soluble fibers (e.g., pectin, inulin, β-glucans) are readily fermented and typically increase the relative proportion of butyrate, whereas insoluble fibers (e.g., cellulose, wheat bran) ferment more slowly and tend to favor acetate production [54].

Other dietary components also modulate SCFA kinetics. High dietary protein levels may promote proteolytic fermentation, generating ammonia and biogenic amines that can inhibit SCFA-producing bacteria. Appropriate dietary fat levels may indirectly influence SCFA metabolism by modulating bile acid secretion and intestinal mucosal function [55].

Environmental factors influence SCFA kinetics by altering both host physiology and microbiota stability. Heat stress represents a common and potent modulator. Under elevated temperatures, redistribution of intestinal blood flow may compromise gut barrier integrity. Beneficial bacteria such as Lactobacillus often decline, whereas opportunistic pathogens such as Escherichia coli increase, leading to impaired fermentation capacity and reduced SCFA production [56,57]. High stocking density acts as a chronic stressor by intensifying competition and spatial restriction, thereby altering gut microbiota composition and function and causing fluctuations in SCFA levels [58]. Moreover, pathogen infections (e.g., *Salmonella* spp. and coccidia) directly induce intestinal inflammation, disrupt microbial homeostasis, reduce populations of SCFA-producing bacteria, and markedly impair SCFA synthesis [59,60]. Additional environmental factors, including photoperiod and ventilation, may also indirectly affect SCFA metabolism through their influence on feeding behavior and digestive physiology.

## 3. Crossing the Barriers: SCFAs as Gut–Brain Messengers—Pathways of Communication

### 3.1. Neural Pathway: How the Vagus Nerve Senses SCFA Signals and Relays Them to the Nucleus of the Solitary Tract, Ultimately Influencing the Hypothalamus

The vagus nerve, the tenth cranial nerve connecting peripheral organs to the central nervous system, is the primary neural pathway in bidirectional gut–brain communication. Its afferent fibers possess abundant terminals within the intestinal mucosa and submucosa, enabling them to keenly sense changes in the gut lumen environment. This allows direct involvement in SCFA-mediated gut–brain signal transmission [61,62]. The biosynthesis, transport, and systemic distribution kinetics of major SCFAs that underpin this signaling are summarized in Table 1.

SCFA activation of the vagus nerve begins with signal perception. Studies indicate that vagal afferent terminals express various receptors capable of sensing SCFAs, most notably G-protein-coupled receptor 41 (GPR41) and G-protein-coupled receptor 43 (GPR43), as demonstrated in rodent models [63]. Although direct evidence for the expression of these receptors in avian vagal neurons remains limited, their presence is inferred from mammalian studies, and their functional role in poultry warrants further investigation. When SCFAs generated in the gut lumen approach the intestinal mucosa via passive diffusion or active transport, they can bind specifically to these receptors. Ligand binding to GPR41 or GPR43 triggers downstream intracellular signaling cascades, including inhibition of adenylate cyclase or activation of phospholipase C. This leads to changes in intracellular second-messenger concentrations, ultimately causing depolarization of the nerve terminal membrane and generation of action potentials, thereby converting the SCFA chemical signal into a neural electrical signal [64,65].

In the signal conduction phase, activated vagal afferent fibers transmit action potentials centripetally along the nerve trunk, with their first central termination point being the nucleus of the solitary tract (NTS) in the medulla oblongata. The NTS is the primary integration center for visceral sensory input and receives most signals from the vagus nerve [66]. Vagal afferents release neurotransmitters such as glutamate onto second-order neurons within the NTS, relaying the SCFA-induced neural signals for initial reception and processing.

This is followed by central signal integration and hypothalamic regulation. After integrating SCFA-related signals from the gut, the NTS forwards this information to the hypothalamus, the higher-order regulatory center for feeding and energy metabolism, primarily through two pathways:

Direct pathway: Specific neuronal populations within the NTS project directly to key hypothalamic nuclei, such as the arcuate nucleus and paraventricular nucleus, thereby delivering signals to feeding-regulatory neurons within these nuclei [67].

Indirect pathway: The NTS influences hypothalamic function indirectly by forming complex neural circuits with other brainstem nuclei [68].

Upon receiving SCFA-related signals from the NTS, the hypothalamus regulates the expression and secretion of key neuropeptides and influences levels of neurotransmitters such as dopamine and serotonin. This ultimately enables precise and rapid regulation of feeding behavior and energy homeostasis in poultry [69]. Studies involving vagotomy have confirmed the necessity of the vagus nerve in this pathway, as the regulatory effects of SCFAs on feeding are significantly attenuated or abolished when vagal transmission is disrupted [70].

### 3.2. Humoral Pathway: How SCFAs Cross the Blood–Brain Barrier to Act Directly on the Central Nervous System

In addition to the neural pathway, the humoral pathway provides another crucial route for SCFAs to transmit gut–brain signals. This pathway relies not on neural electrical signals but on SCFAs being transported via the systemic circulation to the brain, where they cross the BBB and act directly on cells within the CNS [71].

The ability of SCFAs to achieve this relies first on their physicochemical properties. Acetate, propionate, and butyrate are small molecules with moderate lipophilicity. These characteristics allow them to passively diffuse across the lipid bilayer of BBB endothelial cells [72]. The structural basis of the BBB is the tight junctions between brain capillary endothelial cells, which restrict the free passage of hydrophilic substances and large molecules but permit the passage of small, lipophilic molecules via passive diffusion into the brain interstitial space.

In addition to passive diffusion, specific transporter systems expressed on the BBB actively participate in and facilitate trans-barrier transport of SCFAs. Among these, monocarboxylate transporters, particularly MCT1, are key proteins mediating SCFA passage across the BBB [73]. MCT1 is highly expressed on brain capillary endothelial cells. It facilitates the proton-dependent co-transport of SCFAs from the blood into endothelial cells and subsequently releases them from the basolateral side into the brain interstitial space, thereby efficiently delivering SCFAs from the circulation to the brain [74].

Once in the brain interstitial space, SCFAs can directly act on various cell types in the CNS to exert their biological functions:

Astrocytes: As the most abundant glial cells in the brain, astrocytes express high levels of MCT1, GPR41, and GPR43, enabling efficient SCFA uptake. SCFAs can serve as energy substrates fueling for astrocytes via the TCA cycle; additionally, they can modulate the release of neurotrophic factors and inflammatory cytokines by activating receptors, thereby influencing neuronal survival, differentiation, synaptic plasticity, and inflammatory status [75].

Neurons: Some SCFAs can be directly taken up by neurons. Acetate, for instance, can be converted to acetyl-CoA within neurons, participating in energy metabolism and serving as a precursor for the synthesis of the neurotransmitter acetylcholine, thereby regulating neuronal excitability and synaptic transmission [76]. Epigenetic regulation: SCFAs can influence neuronal gene expression through epigenetic mechanisms, particularly via butyrate-mediated inhibition of histone deacetylases, thereby contributing to long-term regulation of physiological functions such as feeding and mood [77].

### 3.3. Immune Pathway: SCFAs Transmit Anti-Inflammatory/Pro-Inflammatory Signals to the Brain Indirectly by Modulating Peripheral Immune Cells and Cytokines

The immune pathway provides an indirect route for SCFA-mediated “gut–brain” communication. In this pathway, SCFAs modulate the peripheral immune system, altering immune cell activity and cytokine secretion profiles. These immune changes then relay anti- or pro-inflammatory signals to the CNS via immune–neural interactions, thereby influencing brain function [78,79]. This process operates primarily at three levels: gut mucosal immunity, systemic immunity, and central transmission of immune signals.

At the gut mucosal immune regulation level: The gut, as the body’s largest immune organ, contains abundant immune cells such as macrophages, dendritic cells, and T lymphocytes in the mucosal layer. SCFAs, particularly butyrate, are crucial regulators of gut mucosal immunity [80]. Their mechanisms include the following:

Epigenetic regulation: Butyrate is a potent inhibitor of histone deacetylases. By inhibiting HDAC activity in immune cells, it increases histone acetylation levels and alters the expression of genes related to cell activation and differentiation. For example, butyrate can inhibit dendritic cell maturation, reducing the expression of surface co-stimulatory molecules and MHC class II molecules, thereby impairing their ability to activate T cells and preventing excessive immune responses [81,82].

Receptor-mediated regulation: SCFAs activate GPR41 and GPR43 on gut immune cells, modulating intracellular signaling pathways and influencing cytokine secretion. For instance, acetate and propionate can activate GPR43 on macrophages, inhibiting the production of pro-inflammatory cytokines while promoting secretion of the anti-inflammatory cytokine IL-10, thereby maintaining gut immune homeostasis [83,84].

At the systemic immune regulation level, SCFAs absorbed into the portal circulation that escape hepatic metabolism can distribute throughout the body via the systemic circulation, thereby modulating immune cell function in peripheral immune organs [85]. SCFAs promote the differentiation and proliferation of regulatory T cells (Treg cells), which are key to maintaining immune tolerance and suppressing excessive inflammation, primarily through secretion of anti-inflammatory cytokines such as IL-10 and TGF-β [86]. SCFAs enhance the generation and function of Treg cells by promoting histone acetylation at the *Foxp3* gene locus via HDAC inhibition [87]. Additionally, SCFAs can inhibit excessive activation of inflammatory cells such as neutrophils, further modulating the intensity of systemic inflammatory responses.

At the central nervous immune signal transmission level, cytokines produced by the peripheral immune system can convey signals to the CNS through several pathways:

Active transport across the BBB: Specific cytokines can be actively transported across the BBB via specialized transporters expressed on brain endothelial cells [88]. Via circumventricular organs: These brain regions have a leaky or incomplete BBB, allowing blood-borne cytokines to passively diffuse into brain tissue and influence nearby brain regions such as the hypothalamus [89].

Activation of vagal afferent fibers: Vagal afferent terminals also express cytokine receptors. When peripheral inflammation elevates cytokine levels, cytokines can activate the vagus nerve, converting an immune signal into a neural signal relayed to the NTS and subsequently influencing the hypothalamus and other brain regions [90].

**Figure 2:**
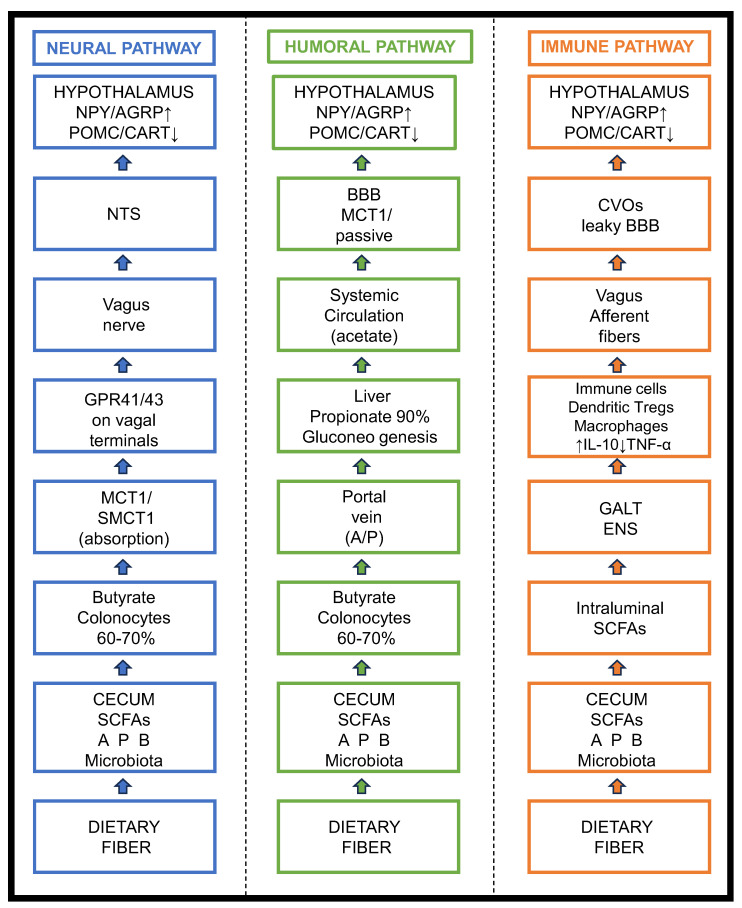
Schematic diagram of the microbiota–gut–brain axis illustrating three SCFA-mediated communication pathways. Dietary fiber is fermented by gut microbiota in the cecum to produce short-chain fatty acids (SCFAs: acetate, propionate, butyrate), which act through neural, humoral, and immune pathways to regulate hypothalamic feeding centers. (1) Neural pathway (**left**): SCFAs activate GPR41/43 receptors on vagal afferent terminals. Signals are transmitted via the vagus nerve to the nucleus of the solitary tract (NTS) and then to the hypothalamus, modulating neuropeptide expression (↑*NPY*/*AgRP*, ↓*POMC/CART*). (2) Humoral pathway (**center**): SCFAs are absorbed across the intestinal epithelium via MCT1/SMCT1 transporters. Butyrate is primarily metabolized by colonocytes (∼60–70%) as an energy source. Acetate and propionate enter the portal vein; propionate undergoes extensive hepatic extraction (∼90%) for gluconeogenesis, while acetate enters systemic circulation and crosses the blood–brain barrier (BBB) via MCT1-mediated transport and passive diffusion to directly access the hypothalamus. (3) Immune pathway (**right**): SCFAs modulate gut-associated lymphoid tissue (GALT) and the enteric nervous system (ENS), promoting an anti-inflammatory phenotype in immune cells (dendritic cells, Tregs, macrophages) characterized by increased IL-10 and decreased TNF-α production. These signals reach the brain via two routes: (i) through circumventricular organs (CVOs) with a leaky BBB; and (ii) via vagal afferent fibers expressing cytokine receptors. This immune modulation promotes microglial M2 polarization and reduces hypothalamic neuroinflammation. Abbreviations: GPR, G-protein-coupled receptor; MCT1, monocarboxylate transporter 1; SMCT1, sodium-coupled monocarboxylate transporter 1; NTS, nucleus of the solitary tract; BBB, blood–brain barrier; CVO, circumventricular organ; GALT, gut-associated lymphoid tissue; ENS, enteric nervous system; NPY, neuropeptide Y; *AgRP*, agouti-related peptide; *POMC*, pro-opiomelanocortin; *CART*, cocaine- and amphetamine-regulated transcript; IL-10, interleukin-10; TNF-α, tumor necrosis factor-α. ↓—reduced; ↑—increased.

When SCFAs modulate gut and systemic immunity, leading to increased levels of anti-inflammatory cytokines or decreased levels of pro-inflammatory cytokines in the periphery, this altered immune status is communicated to the CNS via the pathways described above. This, in turn, affects the activity of microglia and astrocytes. For instance, elevated peripheral IL-10 can suppress microglial activation, reducing the production of pro-inflammatory factors within the CNS [91,92]. Through this indirect yet efficient immune pathway, SCFAs translate metabolic information from the gut microbiota into immune signals, indirectly regulating the inflammatory tone and functional state of the CNS and thereby contributing to the fine-tuning of physiological processes such as feeding behavior and stress responses in poultry. The integrated interplay of these neural, humoral, and immune pathways is illustrated schematically in Figure 2, which provides a visual summary of how SCFAs mediate communication along the microbiota–gut–brain axis to regulate hypothalamic feeding centers.

## 4. Arrival at the Command Center: The Molecular Switch-like Role of SCFAs in the Hypothalamus

### 4.1. Receptor-Mediated Rapid Signal Transduction

#### 4.1.1. GPR41/GPR43 Signal Transduction: Coupling to G Proteins, cAMP/PKA, and MAPK/ERK Pathways

The hypothalamus, the central hub for integrating peripheral signals and regulating feeding and energy homeostasis, expresses the SCFA receptors GPR41 and GPR43 on various neurons and glial cells [93]. Binding of SCFAs to these receptors activates downstream heterotrimeric G proteins and intracellular signaling pathways, rapidly altering neuronal excitability and neuropeptide secretion within minutes to tens of minutes. This provides a fast molecular switch for SCFA regulation of hypothalamic function [94].

GPR41 primarily couples to Gi/o-type G proteins, as characterized in rodent and human studies [95]. Upon SCFA binding, the Gαi/o subunit dissociates and inhibits adenylate cyclase activity, leading to decreased intracellular cyclic AMP (cAMP) levels and reduced protein kinase A (PKA) activity. Although this pathway has not been directly demonstrated in avian hypothalamic neurons, the conservation of G-protein signaling across vertebrates suggests a similar mechanism may operate in poultry. Reduced PKA activity affects the phosphorylation state of downstream targets, rapidly regulating immediate-early gene expression. Concurrently, the released Gβγ dimer can activate phospholipase Cβ (PLCβ), catalyzing the hydrolysis of PIP2 to generate IP3 and DAG. IP3 binds to receptors on the endoplasmic reticulum, triggering Ca^2+^ release from intracellular stores, while DAG, in synergy with Ca^2+^, activates protein kinase C (PKC). Increased intracellular Ca^2+^ and PKC activation collectively modulate ion channel activity, neurotransmitter release, and gene transcription, thereby rapidly altering neuronal function [96].

GPR43 primarily couples to Gq/11-type G proteins. SCFA binding activates the Gαq/11 subunit, which directly activates PLCβ, promoting IP3 and DAG generation, Ca^2+^ release, and PKC activation [97]. Additionally, GPR43 activation can indirectly regulate the MAPK/ERK pathway via kinases such as PKC. Activated ERK can translocate to the nucleus and phosphorylate transcription factors, thereby regulating the expression of genes related to feeding and energy metabolism and enabling rapid transcriptional responses to SCFAs [98].

Notably, crosstalk exists between GPR41- and GPR43-mediated pathways. For instance, GPR41 inhibits the cAMP–PKA pathway, whereas GPR43 activates the PLCβ–IP3/DAG pathway, and both can alter intracellular cAMP and Ca^2+^ concentrations. These second-messenger changes can synergistically affect key nodes such as Ca^2+^/calmodulin-dependent protein kinase, which in turn phosphorylates transcription factors such as CREB, enabling synergistic or antagonistic regulation of downstream gene expression. This enables SCFAs to rapidly and precisely regulate hypothalamic neuronal function [99]. An in-depth analysis of these multi-pathway mechanisms is provided in Table 2.

#### 4.1.2. Histone Deacetylase Inhibition: SCFAs (Particularly Butyrate) as Epigenetic Regulators Directly Influencing Hypothalamic Gene Expression

Beyond rapid receptor signaling, SCFAs also function as epigenetic regulators. Butyrate, one of the most potent HDAC inhibitors among SCFAs, induces lasting changes in chromatin structure within hypothalamic neurons by inhibiting HDAC activity, enabling long-term and stable regulation of gene expression, acting as a slow switch for SCFA action [100].

Histone acetylation is a key epigenetic modification dynamically regulated by histone acetyltransferases (HATs) and histone deacetylases (HDACs). HATs add acetyl groups to histone lysine residues, neutralizing their positive charge and relaxing chromatin structure, which promotes transcription factor binding and gene activation. HDACs remove acetyl groups, leading to chromatin condensation and gene repression [101].

Butyrate, as a competitive HDAC inhibitor, penetrates the cell and nuclear membranes, binds to the catalytic site of HDACs, and blocks their deacetylase activity. It potently inhibits class I and class IIa HDAC members in mammalian cells [102]. Direct evidence for HDAC inhibition by butyrate in avian hypothalamic neurons is lacking, but given the evolutionary conservation of HDAC enzymes, a similar mechanism is likely. HDAC inhibition by butyrate in hypothalamic neurons significantly increases acetylation levels at specific histone sites, transforming chromatin from a condensed to a relaxed state and exposing gene promoter regions for transcription machinery binding [103].

This epigenetic reprogramming directly affects the expression of key genes governing feeding behavior and energy metabolism. For example, in the arcuate nucleus:

Increased histone acetylation can promote transcription of the orexigenic neuropeptide genes *NPY* and *AgRP*. Concurrently, it may suppress the expression of anorexigenic neuropeptide genes, *POMC* and *CART*, potentially through complex regulatory networks [104,105]. The resulting changes in *NPY/AgRP* (upregulated) and *POMC/CART* (downregulated) expression enhance feeding drive in downstream circuits.

Furthermore, butyrate may enhance the expression of metabolic hormone receptors in the hypothalamus by increasing histone acetylation at their gene promoters, thereby improving hypothalamic sensitivity to circulating metabolic hormones and optimizing overall energy metabolism regulation [106]. Beyond these direct epigenetic effects, emerging evidence suggests additional layers of complexity in SCFA-mediated hypothalamic regulation.

Emerging evidence suggests crosstalk between GPR41/43-mediated signaling and epigenetic regulation in hypothalamic neurons. Activation of GPR41/43 by SCFAs not only elicits rapid second messenger cascades but also modulates the phosphorylation status of histone-modifying enzymes. For example, GPR43-mediated activation of the MAPK/ERK pathway can phosphorylate and activate the histone acetyltransferase p300, thereby enhancing H3K27 acetylation at the NPY promoter independently of HDAC inhibition [98,103]. Conversely, GPR41-mediated inhibition of cAMP/PKA signaling may reduce the nuclear export of class IIa histone deacetylases (e.g., HDAC4/5) via phosphorylation-dependent mechanisms, thereby influencing their repressive activity at target gene loci [96,99]. This bidirectional interplay between rapid membrane signaling and slower epigenetic programming enables SCFAs to integrate short-term nutritional cues with long-term transcriptional adaptations, ensuring precise and sustained regulation of feeding behavior.

In addition to direct HDAC inhibition, butyrate influences hypothalamic neuronal function through metabolic reprogramming. As an energy substrate, butyrate enters the tricarboxylic acid (TCA) cycle in mitochondria, increasing the production of acetyl-CoA and α-ketoglutarate. Elevated acetyl-CoA serves as a substrate for histone acetyltransferases (HATs), promoting global histone acetylation independently of HDAC inhibition [103,106]. Meanwhile, α-ketoglutarate acts as an essential cofactor for Jumonji C-domain-containing histone demethylases, potentially facilitating the removal of repressive histone marks such as H3K9me3 and H3K27me3 at specific gene loci [101]. This metabolic–epigenetic coupling allows SCFAs to directly link cellular energy status to chromatin remodeling, providing an additional layer of regulatory control over hypothalamic neuropeptide expression and feeding behavior. This epigenetic regulation by SCFAs is tissue- and gene-specific and works in concert with other modifications such as DNA methylation, forming the basis for long-term programming of hypothalamic function. A systematic analysis of these signaling pathways, from classical to emerging mechanisms, is presented in Table 3.

### 4.2. Energy Sensing and Fine-Tuning of Feeding Behavior

#### 4.2.1. Crosstalk with the AMPK/mTOR Energy-Sensing Pathways

Hypothalamic neurons acutely sense the body’s energy status. AMP-activated protein kinase (AMPK) and the mechanistic target of rapamycin (mTOR) are two core cellular energy sensors. SCFAs engage in crosstalk with the AMPK/mTOR pathways, allowing the hypothalamus to integrate metabolic signals from the gut microbiota and finely tune feeding behavior [107,108].

AMPK is activated under low-energy conditions. SCFAs influence hypothalamic AMPK activity through the following: Epigenetic mechanisms: Butyrate, via HDAC inhibition, can upregulate the expression of LKB1, an upstream kinase of AMPK, thereby promoting AMPK phosphorylation and activation [109]. Receptor and calcium signaling: SCFAs activating GPR41/GPR43 can influence intracellular Ca^2+^ levels, potentially activating CaMKKβ, another upstream kinase that phosphorylates and activates AMPK [110]. Activated AMPK phosphorylates downstream targets to promote catabolism for energy production and can stimulate feeding by regulating hypothalamic neuropeptides, thereby responding to energy deficit.

mTOR is activated when energy and nutrients are abundant. SCFAs regulate the mTOR pathway via the following: Signal activation: SCFAs activating GPR43 and the subsequent PLCβ–IP3/DAG–Ca^2+^ pathway can elevate intracellular Ca^2+^, which may activate Rheb, an upstream regulator of mTORC1, promoting its activation [111].

Epigenetic regulation: Butyrate, through HDAC inhibition, may enhance histone acetylation at the promoters of mTOR or related pathway components, thereby potentiating mTORC1 signaling. Activated mTORC1 promotes anabolism and suppresses feeding by inhibiting *NPY*/*AgRP* and promoting *POMC* expression, coordinating energy storage and expenditure.

The AMPK and mTOR pathways are mutually antagonistic, forming a precise energy-sensing balance. SCFAs, reflecting gut energy availability through their concentration and composition, dynamically adjust this balance: favoring AMPK activation at lower levels to promote feeding and shifting towards mTOR activation at higher levels to limit energy intake. This mechanism supports the maintenance of energy homeostasis [112].

#### 4.2.2. Differential Regulation of Key Neuropeptides: Upregulating *NPY*/*AgRP* and Downregulating *POMC*/*CART*

The coexisting *NPY*/*AgRP* and *POMC*/*CART* neuronal populations in the arcuate nucleus function as the key accelerator and brake for feeding behavior, respectively. Substantial evidence indicates that SCFAs can differentially regulate these neuropeptides, upregulating the expression and secretion of *NPY*/*AgRP* while downregulating *POMC*/*CART*, thereby promoting feeding behavior in the short term [113,114].

Mechanisms for upregulating *NPY*/*AgRP*:

Receptor pathway: SCFAs acting on GPR41 on *NPY*/*AgRP* neurons inhibit the AC–cAMP–PKA pathway via Gi/o. Reduced PKA activity leads to decreased phosphorylation of CREB, which may relieve transcriptional restraint on *NPY*/*AgRP* genes, thereby promoting their transcription [115]. Simultaneously, the Gβγ subunit activates PLCβ, which through the IP3/Ca^2+^ and DAG/PKC pathways activates transcription factors such as AP-1, further promoting *NPY*/*AgRP* gene expression [96].

Epigenetic pathway: Butyrate, via HDAC inhibition, increases histone acetylation at the *NPY* and *AgRP* promoter regions, opening chromatin structure and facilitating the binding of transcription factors, thereby enhancing gene transcription [104].

Mechanisms for downregulating *POMC*/*CART*:

Receptor pathway: SCFAs acting on GPR43 on *POMC* neurons activate the PLCβ–IP3/DAG–Ca^2+^/PKC pathway via Gq/11. Activated PKC can phosphorylate STAT3; phosphorylated STAT3 may then bind to negative regulatory elements on the *POMC* promoter, inhibiting transcription [116]. GPR43 activation might also phosphorylate Elk-1 via the MAPK/ERK pathway, competitively inhibiting binding of other transcriptional activators to the *POMC*/*CART* promoters.

Epigenetic and indirect pathways: Although HDAC inhibition often promotes gene expression, in the context of *POMC*/*CART* regulation, butyrate may enhance expression of certain repressive transcription factors by increasing histone acetylation at their loci, indirectly suppressing *POMC*/*CART* transcription [117]. SCFAs may also induce repressive histone modifications at the *POMC* promoter region, collectively contributing to gene silencing.

This fine-tuned differential regulation ensures that when poultry consume fiber-rich diets and the gut produces substantial SCFAs, the hypothalamus receives an energy substrate availability signal. It responds by moderately promoting feed intake to support energy acquisition while optimizing longer-term metabolic balance to reduce the risk of obesity.

### 4.3. The Fire-Extinguisher Role in Neuroinflammation

#### 4.3.1. Suppressing Activation of the Hypothalamic NF-κB Pathway

Hypothalamic neuroinflammation disrupts feeding regulatory function. The NF-κB pathway is a master regulator of inflammatory responses; its overactivation drives excessive production of pro-inflammatory cytokines, impairing neuronal function and contributing to leptin resistance [118]. SCFAs, particularly butyrate and propionate, have been demonstrated to inhibit hypothalamic NF-κB signaling, thereby exerting a fire-extinguisher effect [119,120].

**Table 2:** In-depth Analysis of Multi-pathway Mechanisms for SCFA-Mediated Gut–Brain Axis Communication.

Core Dimensions & Specific Points	Neural Pathway	Humoral Pathway	Immune Pathway	References
1. Signal Perception & Decoding				
Key Receptors/Transporters	GPR41 (Gi/o), GPR43 (Gi/o, Gq), 5-HT3	MCT1, SMCT1, Passive	GPR43, GPR109A, HDACs	[63,73,80,81,82,83,84]
Ligand Preference & Affinity	GPR41: Prop ≈ But > Acet; GPR43: Prop > Acet > But; 5-HT3: 5-HT	Acet > Prop > But; regulated by pH	GPR109A: But; HDAC: But > Prop > Acet	
Subcellular Localization & Microdomain	Vagal terminal; neuro-endocrine unit	Enterocyte membrane; brain endothelial	Immune cell membrane/nucleus	
2. Intracellular Signal Transduction Cascade				
Second Messengers & Kinases	↓cAMP/PKA, ↑IP_3_/DAG/PKC, ↑intracellular Ca^2+^, MAPK/ERK activation	Formation of transmembrane H^+^/Na^+^ gradients, changes in intracellular Acetyl-CoA/ATP/NADH levels	↓cAMP/PKA (GPR43/Gi), ↑IP_3_/Ca^2+^ (GPR43/Gq), NF-κB/MAPK pathway inhibition	[64,65,78,79,85,86,87]
Ion Channels & Electrophysiology	Voltage-gated Ca^2+^ channel opening, K^+^ channel inhibition, action potential initiation	Indirectly affects neuronal excitability via altering energy metabolism; no direct electrical signal	Modulates immune cell Ca^2+^ signaling, affects cytokine secretion; no direct neural electrical signal	
Transcriptional Regulation Nodes	Rapid expression of immediate early genes (e.g., c-Fos) in NTS	Altered activity of metabolic sensing transcription factors (e.g., ChREBP, PPARs); global increase in histone acetylation	Activation of anti-inflammatory transcription factors (e.g., STAT3, AhR); inhibition of pro-inflammatory factor (e.g., NF-κB) nuclear translocation	
3. Cross-Cellular Communication & Interface				
Cell–cell Dialogue	Enteroendocrine cell → (5-HT/CCK) → Vagal afferent; Vagus nerve → (ACh) → Gut immune cells	Enterocyte → (SCFAs) → Portal circulation; Astrocyte → (lactate/glutamine) → Neuron	Dendritic cell → (IL-10/TGF-β) → T cell; Treg → (IL-10) → Macrophage	[61,62,66,71,72,74,88,89,90]
Biological Barrier Crossing	No need to cross cellular barriers, direct transmission via electrical signals	Must sequentially cross gut epithelial barrier and blood–brain barrier; efficiency determines signal strength	Immune cell migration; cytokines enter brain via “leaky” circumventricular organs	
Functional Synapses/Junctions	Glutamatergic chemical synapses between vagus nerve and NTS neurons	Physical barrier and selective channel formed by BBB endothelial tight junctions	“Immunological synapses” form between immune cells and neurons/glia for information exchange	
4. Central Targeting & Integration				
Primary Central Relay Station	Nucleus of the Solitary Tract (NTS, visceral sensory gateway)	Hypothalamic Arcuate Nucleus (near BBB), periventricular regions	Circumventricular Organs (e.g., Area Postrema), Meninges, Choroid Plexus	[67,68,69,75,76,77,91,92]
Higher-Order Integration Centers	Hypothalamus (ARC, PVN, LH), Parabrachial Nucleus, Amygdala	Hypothalamus (energy sensing), Hippocampus (learning/memory), Cortex (cognition)	Hypothalamus (neuroendocrine center), Amygdala (emotion)	
Specific Cellular Responses	*NPY/AgRP* neurons (activated), *POMC* neurons (inhibited), NTS projection neurons (integration)	Astrocytes (metabolic support), hypothalamic glucose-sensing neurons (excitability changes)	Microglia (phenotype polarization), hypothalamic neurons (reduced inflammatory damage)	
5. Neural Circuits & Information Flow				
Information Flow Direction & Speed	Unidirectional afferent (gut → brain), millisecond-second scale, topographically organized	Diffuse distribution, minute-scale, concentration-dependent, influenced by systemic circulation	Multi-directional, network-based, hour-scale, possesses “immune memory” characteristics	[61,62,63,70,78,79,90]
Circuit Hierarchy	Brainstem → Hypothalamus → Limbic system → Cortex, hierarchical processing	Parallel processing: multiple brain regions receive signals simultaneously for integration	Modulatory input: alters functionality of existing neural circuits by changing the microenvironment	
Feedback Regulation Mechanisms	Satiety signals ascend via same pathway to terminate feeding; hypothalamic-autonomic output regulates gut function	Central regulation of appetite/behavior alters SCFA substrate intake (long-loop feedback)	Central anti-inflammatory signals descend via cholinergic anti-inflammatory pathway to modulate gut immunity	
6. Epigenetic Reprogramming				
Histone Modifications	H3K9ac modification of neuropeptide genes in vagal ganglia may regulate long-term sensitivity	Significantly increased H3K9/K27ac at promoters of hypothalamic *POMC, NPY* genes, persistently altering transcription	Histone hyperacetylation at *Foxp3* locus in T cells and *Il10* promoter in microglia, stabilizing anti-inflammatory phenotype	[77,87,100,101,102,103]
DNA Methylation	-	Altered DNA methylation at promoters of hypothalamic metabolic genes (e.g., LeptinR)	Demethylation at inflammation-related gene regions in immune cells (e.g., TSDR in Tregs)	
Non-coding RNA Networks	Altered miR-143/145 expression in vagus nerve may modulate receptor sensitivity	Regulation of metabolism-related circRNA expression (e.g., Cdr1as) in astrocytes	Upregulation of miRNAs (e.g., miR-10a) in Tregs, suppressing inflammatory cytokine production	
7. Physiological Functional Output				
Rapid Behavioral Adjustment	Immediate feeding initiation/cessation, food-seeking behavior, gastrointestinal reflexes (e.g., gastric emptying)	(Not directly involved in rapid behavior.) Provides metabolic context for behavior.	(Not directly involved in rapid behavior.) Indirectly acts by influencing emotional state.	[16,25,69,70,75,76,91,92]
Energy Metabolism Regulation	Short-term appetite control, meal-by-meal fine-tuning of energy intake	Long-term energy set-point programming, systemic energy allocation and storage regulation	Maintains metabolic homeostasis, prevents energy waste from inflammation (e.g., sickness behavior)	
Neuroimmunity & Protection	Indirectly modulates peripheral inflammation via cholinergic anti-inflammatory pathway	Enhances neuronal stress resistance by providing energy substrates and epigenetic regulation	Directly suppresses central neuroinflammation, protects hypothalamic neurons from metabolic inflammatory damage	
8. Pathway Interplay & Synergy				
Signal Amplification	Vagal activation can increase intestinal permeability, promoting SCFA absorption (positive feedback)	High SCFA concentrations directly suppress appetite via humoral pathway, synergizing with neural signals	Anti-inflammatory environment ensures normal function of neurons/glia, enabling more precise responses to SCFAs	[61,62,63,70,78,79,90,119]
Signal Complementarity	Provides spatiotemporally precise, fast signals	Provides sustained, systemic metabolic background signals	Provides defensive background tone, ensuring the former two operate in a stable environment	
Temporal Synergy	Seconds–minutes scale: responsible for initiation and rapid adjustment	Minutes–hours scale: responsible for state maintenance and medium-term adaptation	Hours–days scale: responsible for long-term functional plasticity and system protection	

- no data available; ↓—reduced; ↑—increased.

SCFAs inhibit NF-κB signaling through multiple mechanisms:

Preventing NF-κB nuclear translocation: In the resting state, NF-κB dimers are sequestered in the cytoplasm by inhibitory IκB proteins. Inflammatory stimuli activate IKK, which phosphorylates and targets IκB for degradation, freeing NF-κB to translocate to the nucleus. Butyrate can suppress IKK activity, limiting IκB degradation, and simultaneously upregulate *IκBα* gene transcription via HDAC inhibition, as demonstrated in mammalian immune cells [121]. This dual action retains more NF-κB in the cytoplasm, thereby limiting inflammatory gene transcription in the nucleus. Whether this mechanism operates identically in avian microglia or hypothalamic neurons remains to be determined.

Inhibiting NF-κB transcriptional activity: Even when NF-κB reaches the nucleus, SCFAs can inhibit its transcriptional function. They may reduce phosphorylation of the p65 subunit by inhibiting upstream kinases, thereby impairing DNA binding. In addition, butyrate promotes the association of HDAC3 with p65, reducing p65 acetylation, which affects its interaction with transcriptional co-activators and diminishes transcriptional activation [122,123].

Regulating crosstalk signals: SCFA-mediated inhibition of cAMP–PKA via GPR41 can indirectly suppress the MAPK/ERK pathway, which otherwise promotes NF-κB activation. Furthermore, SCFA activation of the PI3K/Akt pathway can promote expression of the anti-inflammatory cytokine IL-10, which feeds back to inhibit IKK, thereby reinforcing an anti-inflammatory loop [124].

#### 4.3.2. Modulating Microglial Function to Maintain Homeostasis of the Neuronal Microenvironment

Microglia, the resident immune cells of the CNS, can amplify hypothalamic neuroinflammation when overactivated. SCFAs modulate microglial function by suppressing pro-inflammatory polarization and promoting a shift towards a neuroprotective phenotype, thereby stabilizing the hypothalamic microenvironment [125].

Inhibiting microglial overactivation: Receptor pathway: Microglia express GPR41/GPR43. SCFA binding to GPR41 inhibits the cAMP–PKA–NF-κB axis via Gi/o and can also reduce MAPK/ERK phosphorylation, collectively suppressing microglial activation and pro-inflammatory cytokine release [126]. Activation of GPR43 via the Gq/11–PLCβ–Ca^2+^ pathway may promote IL-10 expression, thereby providing negative feedback on inflammation.

Metabolic reprogramming: Activated microglia rely heavily on glycolysis. Butyrate, as a metabolic substrate, can promote mitochondrial oxidative phosphorylation, reversing their metabolic phenotype from a pro-inflammatory state to a resting/surveillance-like state and thereby inhibiting inflammatory functions [127].

Promoting a shift towards an anti-inflammatory/repair phenotype: Epigenetic drive: Butyrate, by inhibiting HDACs in microglia, increases histone acetylation at promoters of anti-inflammatory genes, thereby promoting their expression [128]. These genes are markers of the anti-inflammatory phenotype and involved in tissue repair and neuroprotection. PPARγ activation: SCFAs can act as ligands for PPARγ or upregulate its expression via HDAC inhibition. PPARγ activation induces microglial polarization towards an anti-inflammatory phenotype, enhancing their capacity for debris clearance and tissue repair [129].

**Table 3:** Systematic Analysis of SCFA Signaling Pathways in the Hypothalamus: From Classical Pathways to Emerging Mechanisms.

Signaling Pathway/Mechanism	Core Molecular Events & Signal Transduction	Functional Output & Physiological Significance	Key Evidence	Regulatory Specificity & Cellular Localization	References
1. GPR41 Signaling Axis	Gi/o protein coupling → inhibition of adenylate cyclase activity → decreased intracellular cAMP levels → suppression of PKA signaling pathway Gβγ dimer release → activation of phospholipase Cβ → generation of IP_3_ and DAG → mobilization of endoplasmic reticulum Ca^2+^ stores Modulation of transient receptor potential M5 ion channel activity	Rapid promotion of feeding behavior, enhancement of appetite neuropeptide expression, response to intestinal energy metabolic status	Significantly attenuated feeding response to SCFAs in GPR41 gene knockout mice	Primarily expressed in *NPY/AgRP* neurons, vagal ganglion neurons	[63,64,65,94,95,96]
2. GPR43 Signaling Axis	Gq/11 protein activation of phospholipase Cβ pathway, concurrent Gi/o protein inhibition of cAMP signaling Protein kinase Cδ isoform-specific phosphorylation of signal transducer and activator of transcription 3, inhibiting its transcriptional activity Regulation of voltage-gated potassium channel Kv2.1 phosphorylation status	Bidirectional regulation of feeding behavior, maintenance of energy metabolic homeostasis, prevention of hyperphagia	Weakened anorexic effects of SCFAs in *POMC* neuron-specific GPR43 knockout	Mainly distributed in *POMC* neurons, intestinal endocrine cells	[63,64,65,97,98,99]
3. Histone Deacetylase Inhibition	Competitive inhibition of Class I/IIa HDACs by butyrate H3K9/27 hyperacetylation Chromatin remodeling, super-enhancer formation	Epigenetic reprogramming, long-term feeding set point	ChIP-seq demonstrates significant enrichment of H3K9ac at *NPY* gene promoter region	Genome-wide effects, most prominent in energy-sensing neurons	[100,101,102,103,104,105,106]
4. Energy Sensing AMPK Axis	Calcium ion activation of Ca^2+^/calmodulin-dependent protein kinase kinase β, phosphorylating AMPK at threonine 172 Slight reduction in intracellular ATP/AMP ratio, allosteric activation of AMPK Enhanced fatty acid β-oxidation metabolic flux	Simulation of energy-deficient state, promotion of energy acquisition behavior, enhancement of catabolism	Intrahypothalamic injection of AMPK agonist (AICAR) mimics SCFAs’ feeding-promoting effects	Specific expression in ARC nucleus neurons, responsive to energy status changes	[107,108,109,110]
5. mTORC1 Nutrient Sensing Axis	Calcium ion activation of Ras homolog enriched in brain, promoting mTORC1 complex membrane translocation Acetate provides acetyl-CoA, promoting histone acetylation Inhibition of UNC-51 like kinase 1 autophagy pathway	Transmission of nutrient sufficiency signals, limitation of energy intake, promotion of anabolism	Rapamycin pretreatment blocks anorexic effects of high-dose SCFAs	Primarily activated in *POMC* neurons, regulated by nutritional status	[107,108,111,112]
6. Neurotransmitter Metabolic Axis	Acetate conversion to acetyl-CoA catalyzed by acetyl-CoA synthetase Acetylcholine synthesis mediated by choline acetyltransferase Regulation of synaptic vesicle release mechanisms	Fine regulation of synaptic transmission, influence on neural circuitry of feeding decision-making	^13^C-labeled acetate PET imaging shows increased hypothalamic acetylcholine synthesis	Astrocyte-neuron metabolic coupling, presynaptic terminal specificity	[45,76]
7. Reactive Oxygen Species Signaling Axis	Regulation of electron transport efficiency in mitochondrial complexes I and III Moderate mitochondrial ROS activation of Nrf2/ARE pathway Modulation of Keap1-Nrf2 protein interaction	Coupling of metabolic state and electrical activity, bidirectional regulation of neuronal excitability	N-acetylcysteine pretreatment partially attenuates acute feeding-promoting effects of SCFAs	Mitochondrial specificity, most significant in metabolically sensitive neurons	[124]
8. Neuroimmune Regulation Axis	Synergistic inhibition of NF-κB pathway by HDAC inhibition and GPR109A activation Promotion of microglial polarization toward M2 anti-inflammatory phenotype Increased secretion of IL-10 and TGF-β	Maintenance of feeding center microenvironment stability, prevention of metabolic inflammatory damage	Butyrate pretreatment significantly alleviates LPS-induced hypothalamic inflammation	Microglial specificity, blood–brain barrier interface cells	[80,81,82,83,84,119,120,121,122,123,124,125,126,127,128,129]
9. Calcium Signaling Integration Axis	Coordination of GPCR and voltage-gated calcium channel signaling Regulation of endoplasmic reticulum calcium store replenishment and calcium-induced calcium release Activation of nuclear factor of activated T-cells transcription factor family	Integration of multi-pathway signal inputs, maintenance of calcium homeostasis balance	Calcium imaging shows SCFA-induced specific calcium oscillations in hypothalamic neurons	Neuronal soma and dendrite specificity, spatiotemporally specific regulation	[64,65,96,97,98]
10. Cyclic AMP Signaling Axis	Regulation of adenylate cyclase isozyme activity Influence on cAMP response element-binding protein phosphorylation Modulation of exchange protein directly activated by cAMP subtypes	Fine regulation of transcriptional activity, mediation of long-term synaptic plasticity	FRET detection shows SCFAs alter cAMP dynamics	Neuronal postsynaptic density region, nuclear transcription regulation area	[95,96,97,98,99]
11. Autophagy Flux Regulation Axis	Regulation of autophagy initiation complex ULK1/2 activity Influence on microtubule-associated protein 1 light chain 3 lipidation process Control of autophagosome-lysosome fusion efficiency	Maintenance of protein homeostasis, impact on long-term neuronal function	Electron microscopy reveals altered autophagosome numbers in SCFA-treated hypothalamic neurons	Neuronal axon terminals, metabolically sensitive compartments	[111,112]
12. Circadian Rhythm Regulation Axis	Regulation of brain and muscle Arnt-like protein 1 transcriptional activity Influence on Period and Cryptochrome gene expression Control of rhythmic expression of nuclear receptor Rev-erbα	Coordination of feeding rhythm and metabolic cycles, maintenance of circadian clock synchronization	Bioluminescence imaging shows SCFAs alter suprachiasmatic nucleus rhythm	Suprachiasmatic nucleus neurons, core rhythm regulation region	[25,112]

**Figure 3:**
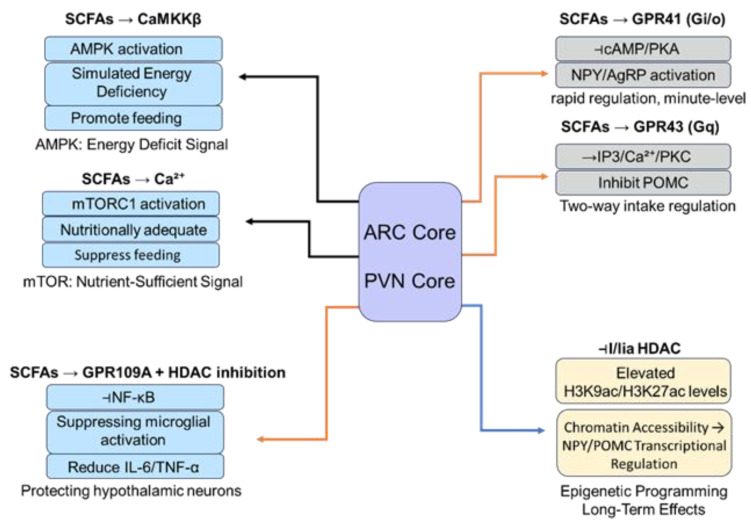
Molecular Mechanisms in the Hypothalamus: Integrated Diagram of Four Core Pathways. SCFAs reaching the hypothalamus act through four interconnected mechanisms to regulate feeding behavior and energy homeostasis: (1) Receptor-mediated signaling—SCFAs activate GPR41 (primarily on *NPY*/*AgRP* neurons) and GPR43 (primarily on *POMC* neurons). GPR41 coupling to Gi/o inhibits cAMP/PKA signaling, while GPR43 coupling to Gq/11 activates PLCβ/IP_3_/DAG pathways, leading to rapid changes in neuronal excitability and neuropeptide expression. (2) Epigenetic regulation—Butyrate inhibits histone deacetylases (HDACs), increasing histone acetylation (e.g., H3K9ac, H3K27ac) at promoter regions. This relaxes chromatin structure, promoting transcription of orexigenic neuropeptide genes (*NPY*, *AgRP*) while indirectly repressing anorexigenic genes (*POMC*, *CART*) through complex regulatory networks. (3) Energy sensing—SCFAs modulate AMPK and mTOR pathways, integrating information about peripheral energy status. Lower SCFA levels favor AMPK activation (promoting feeding), while higher levels shift toward mTOR activation (limiting intake). (4) Neuroimmune modulation—SCFAs inhibit NF-κB nuclear translocation and transcriptional activity, reducing pro-inflammatory cytokine production. They also promote microglial polarization toward the M2 anti-inflammatory phenotype, maintaining hypothalamic microenvironment stability. These pathways operate at different timescales (seconds to hours) and collectively enable precise regulation of feeding behavior. ⊣, inhibition.

SCFA regulation of microglia is dynamic and adaptive, inhibiting excessive activation in early stages to prevent damage and facilitate repair in later stages. This immunomodulation represents an important mechanism by which SCFAs protect hypothalamic function and support metabolic health [130,131,132,133]. The integrated interplay of the four core molecular mechanisms discussed in this chapter is illustrated in Figure 3.

## 5. From Theory to Practice: Harnessing the Power of SCFAs Through Nutritional Strategies

### 5.1. Substrate Engineering: Precision Design of Dietary Fiber Sources (Structure, Solubility, and Degree of Polymerization) to Directionally Modulate the SCFA Profile

Dietary fiber is the primary substrate for microbial fermentation and SCFA production in the gut. Substrate engineering, which involves the precise selection and design of fiber sources and structures, allows for the directional modulation of SCFA yield and composition, analogous to formulating a feed recipe. This is the primary strategy for achieving precision SCFA nutrition [134]. Evidence-based, SCFA-targeted precision nutrition strategies for poultry are compiled in Table 4.

Fiber solubility is a key determinant of fermentation characteristics and the resulting SCFA profile. Soluble fibers (e.g., pectin, β-glucans, inulin, FOS) have loose molecular structures and are rapidly fermented by microbes, often starting in the upper intestinal segments. They significantly increase total SCFA production and often selectively elevate the proportion of butyrate [135]. For instance, inulin, a typical soluble fructan, is preferentially utilized by butyrate-producing bacteria (e.g., *Faecalibacterium prausnitzii*, *Roseburia* spp.), leading to a significantly higher butyrate proportion post-fermentation compared with other fiber sources [136].

Insoluble fibers (e.g., cellulose, wheat bran, lignin) have dense structures and ferment slowly, being degraded predominantly in the distal large intestine. They produce relatively lower total SCFAs, with acetate as the major product (often exceeding 70%). However, their physical properties are crucial for maintaining normal gut motility and improving intestinal morphology [137]. Therefore, in practical diet formulation, balancing the ratio of soluble to insoluble fiber (e.g., between 1:2 and 1:3) can achieve an equilibrium between total SCFAs and their proportions, harnessing the health benefits of SCFAs while avoiding issues such as excessive gas production.

Fiber degree of polymerization influences the kinetics of SCFA production. Low-polymerization-degree fibers (e.g., FOS, GOS; degree of polymerization 2–10) have short molecular chains and are rapidly decomposed, reaching peak SCFA concentrations within 2–4 h post-ingestion. They are suitable for strategies requiring a rapid increase in SCFA levels [138].

High-polymerization-degree fibers (e.g., long-chain inulin, some hemicelluloses; DP > 20) require microbes to secrete more enzymes for gradual breakdown. SCFA production is slower but sustained (up to 12–24 h), favoring long-term stability of SCFA levels in the gut [139]. Based on the physiological stage of the birds (e.g., rapidly growing chicks vs. maintenance in layers) and specific goals (e.g., rapid repair vs. long-term maintenance), scientifically combining fibers with different degrees of polymerization allows for precise temporal control of SCFA generation.

Fiber monomer composition (glucose, fructose, galactose, arabinose, etc.) determines its selectivity for utilization by specific microbes, thereby influencing the SCFA profile [140]. Fructose-based fibers (e.g., inulin) preferentially promote butyrate-producing bacteria; galactose-based fibers (e.g., GOS) tend to enrich bifidobacteria, whose fermentation products are primarily acetate and propionate; arabinoxylan is mainly utilized by Bacteroides and related taxa, generating a higher proportion of acetate. Therefore, precise selection based on fiber monomer composition enables tailored modulation of the SCFA profile.

### 5.2. Microbiota Engineering

#### 5.2.1. Prebiotics: Screening Specific Oligosaccharides That Efficiently Promote SCFA-Producing Microbiota

Prebiotics are selectively indigestible food ingredients that promote the growth and/or activity of one or a limited number of beneficial gut bacteria, thereby improving host health. Screening prebiotics that efficiently and specifically promote SCFA-producing microbiota is a core approach for microbiota engineering [141].

Ideal pro-SCFA prebiotics are typically characterized as high microbial selectivity (primarily utilized by SCFA producers rather than pathogens), high fermentability, good stability in the digestive tract (resisting gastric acid and enzymes to reach the hindgut), and an appropriate dose–response profile [142]. Promising prebiotics in poultry nutrition include the following:

Fructo-oligosaccharides: These can be specifically utilized by bifidobacteria and some butyrate producers, significantly increasing total cecal SCFAs and the proportion of butyrate, often through a cross-feeding mechanism [143].

Galacto-oligosaccharides: These exhibit broader microbial selectivity and can enrich key butyrate producers such as *Faecalibacterium prausnitzii*. They increase total SCFAs while also raising the proportion of propionate [144].

Chito-oligosaccharides: These have both antimicrobial and prebiotic functions, inhibiting pathogens while being utilized by some SCFA producers. They can sustainably elevate SCFA levels, particularly butyrate [145].

Modern screening technologies, such as in vitro fermentation models, metagenomic/metatranscriptomic analyses, and validation in animal trials, have greatly enhanced the precision and efficiency of identifying high-efficacy prebiotics, providing powerful tools for targeted regulation of SCFAs [146].

#### 5.2.2. Probiotics and Synbiotics: Direct Supplementation of Acid-Producing Bacteria or Combination with Prebiotics

Direct supplementation with exogenous probiotics or the use of synbiotics (combinations of probiotics and prebiotics) provides a more direct strategy to rapidly optimize gut microbiota composition and elevate SCFA levels [147].

Effective SCFA-oriented probiotics require strong acid-producing capacity, robust gastrointestinal colonization potential, high safety, and processing stability. Commonly used SCFA-related probiotics in poultry include:

*Lactobacillus* spp. (e.g., *Lactobacillus plantarum*): These primarily produce lactate and acetate. They can also break down complex carbohydrates, providing substrates for butyrate producers (“cross-feeding”) and indirectly promoting butyrate synthesis [148].

*Bifidobacterium* spp. (e.g., *Bifidobacterium longum*): These mainly produce acetate and lactate and can indirectly promote butyrate generation through the “acetate–butyrate cross-feeding” mechanism [149].

*Clostridium butyricum*: This is a direct and efficient butyrate producer. Its spore structure tolerates gastrointestinal challenges, and upon colonization, it can increase cecal butyrate concentration and improve gut barrier function [150].

*Propionibacterium* spp. (e.g., *Propionibacterium acidipropionici*): These are obligate producers of propionate, helping to optimize energy metabolism and reduce fat deposition [151].

Synbiotics, through scientific combinations of probiotics and prebiotics, aim to achieve synergistic effects (often described as “1 + 1 > 2”). The prebiotic provides selective substrates for the supplemented probiotic, promoting its colonization and proliferation in the distal small intestine and cecum. The probiotic, in turn, efficiently ferments the prebiotic, producing more SCFAs and further improving the intestinal microenvironment [152]. For example, the “*Clostridium butyricum* + FOS” synbiotic resulted in significantly greater increases in intestinal *C. butyricum* abundance and butyrate concentration compared with either component alone, and more effectively improved poultry performance [153].

### 5.3. Exogenous Regulators: The Leverage Effect of Phytochemicals (e.g., Chlorogenic Acid, Resveratrol) in Indirectly Boosting SCFAs by Reshaping the Microbiota

Phytochemicals, as a class of natural and safe exogenous bioactive compounds, can act as leverage factors. They are often effective at relatively low doses by remodeling gut microbiota structure, thereby indirectly yet effectively elevating SCFA levels. They serve as an important supplement to SCFA modulation strategies [154].

Chlorogenic acid: Widely found in plants such as coffee and honeysuckle. Its mechanisms for boosting SCFAs include: (1) selectively inhibiting pathogens such as *E. coli* and *Salmonella*, freeing ecological niches for beneficial bacteria; (2) lowering gut pH, creating a favorable environment for acid-producing bacteria; (3) providing metabolites that can serve as substrates for SCFA producers; and (4) enhancing the activity of key microbial enzymes involved in acid production. Studies show that supplementing broiler diets with 200–500 mg/kg chlorogenic acid significantly increases total cecal SCFAs and the proportion of butyrate, while improving gut health and performance [155,156].

Resveratrol: Found in grape skin, Japanese knotweed, and other sources. It may act by (1) increasing gut microbiota alpha diversity and raising the relative abundance of SCFA producers; (2) upregulating microbial functional genes related to carbohydrate metabolism and SCFA synthesis; and (3) exerting synergistic antioxidant and anti-inflammatory effects with SCFAs, stabilizing the gut environment and indirectly supporting the growth of SCFA producers. Supplementation with 50–200 mg/kg resveratrol elevates cecal SCFA levels in broilers and may alleviate performance decline under stress [157,158].

Other phytochemicals, such as tea polyphenols and allicin, may exert similar leverage effects, providing diverse natural options for green poultry farming [159].

### 5.4. Challenges and Considerations: Dose–Effect Relationships, Functional Specificity of Different SCFAs, and the Impact of Individual Microbiota Variations

Despite the promising prospects of modulating SCFAs, several key challenges must be carefully considered in practical application: Dose–effect relationship: The biological effects of SCFAs often follow U-shaped or bell-shaped dose–response patterns. Doses that are too low may be ineffective, whereas excessively high doses may produce adverse effects.

For endogenous production, insufficient dietary fiber leads to SCFA deficiency, but excess fiber may cause digestive disorders or even intestinal epithelial damage. For exogenous supplementation, the optimal dosing ranges for acetate, propionate, and butyrate differ, and the physiological stage of the birds must be considered, requiring precise control [160,161].

Functional specificity: Acetate, propionate, and butyrate, while all SCFAs, have distinct and sometimes divergent physiological roles. Acetate is an important energy source and biosynthetic precursor but can suppress appetite via central mechanisms. Propionate primarily supports hepatic gluconeogenesis, aiding energy metabolism and reducing fat deposition. Butyrate is the preferred energy substrate for intestinal epithelial cells and is crucial for barrier function and anti-inflammatory effects. Ignoring this specificity and focusing solely on total SCFAs may lead to “functional mismatch.” For example, simply increasing butyrate during the finishing period might lead to excessive feed intake, adversely affecting feed conversion ratio [162].

Individual microbiota variation: The unique gut microbiota “fingerprint” of each bird, shaped by breed, age, sex, rearing environment, and health status, can lead to substantial variation in responses to the same nutritional intervention—a “same diet, different effect phenomenon” [163]. For instance, broilers and layers differ in baseline microbiota structures and respond differently to the same fiber source. Chicks have immature microbiota, resulting in lower probiotic colonization efficiency than adults. Diseased birds with dysbiosis may respond poorly to nutritional strategies [164].

Addressing these challenges requires a shift towards precision nutrition, including establishing databases linking poultry microbiota–SCFA profiles–phenotypes; using machine learning to predict individual responses; developing rapid detection technologies; and implementing dynamic nutritional strategies (for example, “base diet + personalized supplementation”) to maximize and stabilize the benefits of SCFA modulation [165,166].

**Table 4:** Evidence-Based SCFA-Targeted Precision Nutrition Strategies for Poultry.

Strategy Dimension	Intervention Category	Specific Protocols	Target SCFA Profile	Application Stage	Research Evidence & Biological Effects	References
Substrate Engineering	Soluble Fiber	Inulin FOS β-Glucans	Significantly increases butyrate proportion	Brooding Period Stress Period	Improves intestinal barrier Modulates hypothalamic neuropeptides Enhances vaccine response Alleviates heat stress decline	[20,21,22,52,56,57,135,136]
	Insoluble Fiber	Wheat Bran Oat Hulls Rice Hulls	Increases acetate proportion	Growing Period Finishing Period	Promotes digestive organ development Regulates lipid metabolism genes Reduces abdominal fat Improves meat quality	[22,53,54,137]
	Resistant Starch	High-amylose starch Modified starch	Sustained and stable butyrate production	Full Production Cycle	Improves insulin sensitivity Affects reproductive hormones Improves eggshell quality Modulates hepatic lipid genes	[22,134]
Microbiota Engineering	Probiotics	*C. butyricum* *L. plantarum* *E. faecalis*	Increases butyrate production	Stress Period Recovery Period	Reduces inflammatory cytokines Improves microbiota diversity Increases antioxidant activity Enhances pathogen clearance	[30,31,32]
	Prebiotics	GOS COS Isomaltooligosaccharides	Modulates SCFA profile	Full Production Cycle	Increases sIgA Modulates cholesterol metabolism Improves tight junctions Enhances vaccine efficacy	[141,142,143,144,145,146]
	Synbiotics	*C. butyricum* + Inulin *L. plantarum* + FOS	Significantly enhances butyrate	Critical Stages	Improves production performance consistency Enhances gut colonization resistance Improves nutrient metabolic utilization Reduces environmental stress impacts	[152,153]
Exogenous Regulators	Polyphenols	Chlorogenic Acid Resveratrol Tea Polyphenols	Increases total SCFAs	Stress Period	Improves oxidative stress Modulates HPA axis Protects intestinal mucosa Improves heat tolerance	[154,155,156,157,158,159]
	Essential Oils	Carvacrol Thymol Cinnamaldehyde	Optimizes SCFA ratio	High-Density Rearing	Improves gut microbial niche Regulates pancreatic digestive enzyme secretion Increases protein digestibility Reduces ammonia emissions	[154,155]
Integrated Application	Precision Programming	Phased protocols Dynamic adjustments	Dynamic balance	Full Production Cycle	Achieves optimal production performance Improves flock health uniformity Enhances product quality consistency Increases comprehensive economic benefits	[134,152,153,160,161,162,163,164,165,166]

## 6. Limitations and Knowledge Gaps in Poultry SCFA Research

While this review highlights the central role of SCFAs in regulating feed intake via the MGB axis, it is important to acknowledge several limitations and knowledge gaps that temper the direct translation of these findings to poultry.

### 6.1. Extrapolation from Mammalian Studies: A Necessary but Cautious Approach

Much of our current understanding of the molecular mechanisms underlying SCFA signaling—particularly regarding receptor-mediated pathways, epigenetic regulation, and neuroimmune modulation—is derived from mammalian models, primarily rodents and humans. Although these studies provide a valuable conceptual framework, direct experimental validation in avian species remains limited. Throughout this review, we have explicitly noted where findings are inferred from mammalian research. However, it must be emphasized that the extent to which these mechanisms operate identically in poultry warrants further investigation. Key differences in neuroanatomy, metabolism, and immune function between birds and mammals may lead to species-specific variations in SCFA signaling.

### 6.2. Species-Specific Differences in Neuroanatomy and Metabolism

Birds possess unique neuroanatomical features that distinguish them from mammals. For instance, the avian hypothalamus, while functionally analogous to its mammalian counterpart, exhibits distinct cytoarchitecture and neuropeptide distribution patterns. Similarly, the avian blood–brain barrier and circumventricular organs differ in their permeability and transporter expression profiles, potentially affecting SCFA access to central feeding centers. Metabolic differences also exist: poultry have higher basal metabolic rates, distinct hepatic gluconeogenic pathways, and different patterns of lipid metabolism compared with mammals. These species-specific traits may influence how SCFAs are processed and how they exert their biological effects.

### 6.3. Methodological Challenges in Poultry Hypothalamic Research

Investigating hypothalamic mechanisms in poultry presents several methodological challenges. First, the small size of the avian brain makes targeted microinjection and electrophysiological recording technically demanding. Second, there is a lack of poultry-specific antibodies and molecular tools for detecting receptors, signaling molecules, and epigenetic modifications. Third, most studies to date have measured SCFA concentrations in cecal contents or peripheral blood, with limited data on SCFA levels in the cerebrospinal fluid or hypothalamic tissue. Fourth, dose–response relationships for SCFAs in poultry remain poorly characterized, making it difficult to establish optimal dietary supplementation strategies.

### 6.4. The “One-Bird-One-Strategy” Concept: Vision or Reality?

The concept of precision feeding—tailoring nutritional interventions to individual birds based on their microbiota and metabolite profiles—represents an ambitious long-term vision. While advances in high-throughput sequencing, rapid metabolite detection, and machine learning are bringing this goal closer, significant practical hurdles remain. Commercial poultry production typically involves large flocks where individual monitoring is impractical. Moreover, the cost of personalized nutritional strategies currently exceeds the economic margins of poultry farming. Nevertheless, we envision a stepwise progression: from population-level recommendations to group-specific strategies (e.g., based on breed, age, or health status), and ultimately to individualized approaches as technology advances and costs decrease. The “one-bird-one-strategy” concept should therefore be viewed as an aspirational target that guides research directions rather than an immediately achievable practice.

## 7. Conclusions and Perspectives

This review has systematically outlined the central role of short-chain fatty acids (SCFAs) within the Microbiota–Gut–Brain (MGB) axis, demonstrating that SCFAs function as indispensable chemical messengers integrating the diet–microbiota–gut–brain signaling network.

SCFAs as integrators. Dietary fiber provides fermentation substrates for gut microbiota, which convert fiber into SCFAs via specific functional genes. SCFAs then act through three coordinated pathways—neural (vagus nerve), humoral (blood-borne delivery), and immune (cytokine modulation)—to encode gut metabolic status into signals decipherable by the central nervous system [61].

Hypothalamic mechanisms. Upon reaching the hypothalamus, SCFAs exert dual control over feeding behavior: (i) rapid receptor-mediated signal transduction via GPR41/GPR43, modulating cAMP/PKA and PLCβ/IP_3_/DAG pathways; and (ii) slow epigenetic programming through HDAC inhibition, particularly by butyrate, which alters histone acetylation at neuropeptide gene promoters [100,104]. SCFAs also function as neuroinflammatory modulators, inhibiting NF-κB signaling and promoting microglial M2 polarization, thereby maintaining hypothalamic microenvironment stability [119,125].

Nutritional strategies. Practical approaches to enhance SCFA production include substrate engineering (precision design of fiber sources), microbiota modulation (probiotics, prebiotics, synbiotics), and exogenous regulators (phytochemicals such as chlorogenic acid and resveratrol). However, dose–effect relationships, functional specificity of individual SCFAs, and individual microbiota variation must be carefully considered [160,163].

Limitations. As acknowledged in Section 6, much of our mechanistic understanding is derived from mammalian studies, and direct validation in poultry remains limited. Species-specific differences in neuroanatomy, metabolism, and immune function warrant further investigation.

Future directions. Key research priorities include the following: (i) utilizing multi-omics technologies to unravel the global molecular network of SCFA regulation; (ii) developing novel feed additives such as SCFA receptor agonists or slow-release formulations; (iii) establishing predictive models for precision nutrition based on microbiota and SCFA profiles; and (iv) investigating transgenerational epigenetic programming by SCFAs [167,168,169,170,171,172,173].

Industrial implications. Deepening our understanding of SCFAs is driving a paradigm shift in poultry nutrition—from a traditional focus on meeting growth needs toward actively programming health through MGB axis modulation. This transformation promises to reduce antibiotic reliance, enhance production stability, improve product quality, and ultimately propel the poultry industry toward sustainable, high-quality development [174]. Key remaining unknowns and cutting-edge solutions in SCFA research are summarized in Table 5.

## Figures and Tables

**Table 1:** Biosynthesis, Transport, and Systemic Distribution Kinetics of Major SCFAs in Poultry.

Property	Acetate	Propionate	Butyrate	References
1. Basic Properties & Proportion				
Molecular Weight (Da)	60.05	74.08	88.11	[33,47]
Relative Proportion in Cecum (%)	50–70%	15–25%	10–20%	
pKa (Governs Ionization)	4.76	4.87	4.81	
2. Synthesis Niche & Pathways				
Precursor Substrate Preference	Universal sugar fermentation (glucose, xylose); lactate utilization. Broad ecological niche.	Succinate conversion (Bacteroidetes-dominated); lactate disproportionation; amino acid fermentation (enhanced under protein excess).	Specific polysaccharide fermentation (resistant starch, arabinoxylan); relies on cross-feeding (uses acetate, lactate). Specialized, strict anaerobic niche.	[34,35,36]
Core Biosynthetic Pathways	Pta-AckA; Wood-Ljungdahl	Succinate; Acrylate	But-CoA transferase; Butyrate kinase	
Key Regulatory Factors	Substrate type, H_2_ pressure	Substrate (sugars/lactate), gut pH	Genetics, acetate, pH, O_2_	
3. Producer Community & Function				
Dominant Cecal Producers in Poultry	*Bacteroides* spp. (primary polysaccharide degraders); *Bifidobacterium* (also produces lactate); *Akkermansia muciniphila* (mucin degrader).	*Bacteroides* spp. (via succinate pathway); *Phascolarctobacterium* (obligate utilizer of lactate/succinate); *Megasphaera elsdenii* (abundance increases under stress).	*Faecalibacterium prausnitzii* (anti-inflammatory); *Roseburia spp.* (key dietary fiber degrader); *Clostridium butyricum* (probiotic strain); *Eubacterium rectale*.	[30,31,32]
4. Absorption & Portal Kinetics				
Intestinal Absorption Mechanism	Passive diffusion + MCT1/SMCT1	MCT1-mediated	MCT1; 60–70% oxidized in colonocytes	[38,39,40,41,47]
Portal Vein Concentration (μM)	400–1200	100–400	50–150	
5. Systemic Distribution & Metabolic Fate				
Peripheral Blood Concentration (μM)	50–200	10–50	1–10 (Very low)	[42,43,44,45,46,47]
Primary Metabolic Organs/Cells	Liver, muscle, adipose	Liver (gluconeogenesis)	Colonocytes (fuel), Liver (minor)	
Core Metabolic Fate & Role	Circulating energy currency; converted to acetyl-CoA for TCA/lipogenesis	Liver glucose precursor; 90% extracted for gluconeogenesis	Colonocyte fuel; HDAC inhibition in periphery	
6. Blood–brain barrier & Central Action				
Blood–brain barrier Permeability	High (MCT1-mediated + passive diffusion)	Moderate (MCT1-mediated)	Low (Only when BBB permeability is increased)	[44,45,46]
Primary Target Cells in CNS	Astrocytes, Neurons	Neurons, Microglia	Microglia, Neurons	
Core Central Function & Mechanism	Astrocytes → acetyl-CoA for energy, ACh, histone acetylation	Neurons, microglia; neuroinflammatory modulator	Microglia, neurons; HDAC inhibition, anti-inflammatory	

**Table 5:** Key Unknowns and Cutting-Edge Solutions in SCFA Research.

Key Scientific Questions/Technical Bottlenecks	Limitations of Current Technologies	Future Feasible Technical Pathways	Expected Outcomes and Deliverables	Potential Impact on Poultry Research and Industry	References
Spatiotemporal Dynamics and Rhythmic Monitoring of SCFAs	Relying on endpoint measurements fails to capture the dynamic changes in SCFAs within living organisms in response to diet and circadian rhythms.	Fluorescent probes Wearable biosensors MALDI-MSI imaging	Revealing the “metabolic rhythm index” of SCFAs Establishing a dynamic precision nutrition intervention timing model Analyzing the distribution gradient of SCFAs in tissue and cellular microenvironments	Achieve “Chrono-specific Precision Nutrition” aligned with natural feeding rhythms Optimize feeding schedules to maximize SCFA health benefits Revolutionize the paradigm for evaluating feed additive efficacy	[47,48,169,170]
Cell-Type and Neural Circuit-Specific Mechanisms of SCFAs	The lack of avian-specific genetic manipulation tools makes it difficult to elucidate the specific roles of SCFAs in different neuronal subtypes and glial cells.	Single-cell multi-omics Organoids Spatial transcriptomics	Construct a refined “SCFA-Neuron Type-Function” map Identify key neural circuits mediating SCFA effects Discover novel cell-type-specific receptors or co-factors	Develop neuro-targeted additives to precisely regulate specific behaviors (e.g., feeding, stress) Avoid side effects of nutritional interventions, improving welfare and production stability	[69,75,76,77,169,170]
SCFA Crosstalk within the Gut–Brain Axis Signaling Network	Research often focuses on single SCFAs or pathways, lacking integration of SCFAs with other signals like bile acids and tryptophan metabolites.	Spatial multi-omics SCFA interactome mapping Organ-on-a-chip	Reveal the hub role of SCFAs in the gut–brain axis signaling network Discover key nodes for pathway synergy/antagonism Elucidate reprogramming of signaling networks under different dietary structures	Design multi-target, synergistic compound feed additives Systematically enhance poultry health through combined nutritional strategies, reducing single additive dosage	[61,62,63,64,78,79,90,169,170]
Predicting Individual SCFA Response & Precision Nutrition	Inability to predict individual bird’s response to SCFA intervention based on microbiome features, leading to “non-responders”.	Cohort studies Machine learning models Gene-guided nutrition	Establish a prediction system for SCFA nutritional response based on baseline microbiome Identify microbial biomarkers for “high responders” Achieve dynamic, precise nutritional recommendations (“one-bird-one-strategy”)	Significantly improve feed utilization efficiency, reducing ineffective inputs and costs Enhance flock uniformity, boosting overall production performance and economic returns	[163,164,165,166,173]
SCFA-Mediated Mechanisms of Behavior and Welfare Regulation	Disconnect between behavioral phenotyping and molecular mechanisms; lack of direct causal evidence linking SCFAs to specific behaviors (e.g., feather pecking, fear).	Behavioral monitoring + neural recording Optogenetics/chemogenetics Neural tracing	Establish causal links: “SCFA → Specific Neural Circuit → Behavioral Output” Decipher the neural mechanisms by which SCFAs alleviate stress and improve welfare Elucidate SCFA effects on poultry cognition and social behavior	Develop nutritional strategies to effectively mitigate behavioral disorders and improve welfare Reduce behavioral issues in farming through nutritional regulation, ensuring sustainable industry development	[15,17,18,26,69,119]
Transgenerational Epigenetic Programming by SCFAs	Limited understanding of how SCFA-induced epigenetic changes are transmitted and affect offspring health (e.g., disease resistance, stress resilience).	Single-cell multi-omics Genome editing Multi-generation models	Map transgenerational epigenetic modifications induced by SCFAs Identify critical windows for parental nutritional programming affecting offspring health Determine the stability and reversibility of SCFA epigenetic effects	Programmatically enhance offspring’s lifelong health and performance through parental nutrition Breed flocks with superior “metabolic memory,” fundamentally enhancing industry resilience	[100,101,102,103,104,105,106,169,170]
SCFA-Immune System Dialogue Mechanisms	There is insufficient understanding of the molecular details of how SCFAs specifically regulate the differentiation and function of avian immune cells, such as mucosal Tregs.	Lineage tracing Organoid-immune co-culture Modified SCFA molecules	Elucidate unique pathways by which SCFAs regulate avian mucosal immune homeostasis Discover mechanisms for SCFA’s adjuvant effects enhancing vaccine responses Develop SCFA-derived formulations targeting immunometabolism	Develop immunometabolic modulators as antibiotic alternatives for precise gut disease control Enhance vaccine efficacy, establishing a new frontier for disease prevention	[80,81,82,83,84,85,86,87,91,92,119,120,121,122,127,128]
Precise Regulation of Microbial SCFA Synthesis	Inability to directionally enhance specific SCFA ratios within complex microbiota or achieve targeted enrichment in specific gut segments.	Synthetic biology Phage therapy Metabolic engineering	Develop “Live Biotherapeutic Products” that produce high yields of specific SCFAs (e.g., butyrate) Establish spatiotemporally specific systems for regulating SCFA synthesis Achieve personalized customization of SCFA profiles	Precisely shape an ideal gut micro-ecological and metabolic environment Give rise to “next-generation probiotics/synbiotics” for precise gut health management	[37,38,39,141,142,143,144,145,146,147,148,149,150,151,152,153,171,172]

## Data Availability

No new data were created or analyzed in this study. Data sharing is not applicable to this article.
